# Systematic review and meta-analysis of the prevalence of chronic fatigue syndrome/myalgic encephalomyelitis (CFS/ME)

**DOI:** 10.1186/s12967-020-02269-0

**Published:** 2020-02-24

**Authors:** Eun-Jin Lim, Yo-Chan Ahn, Eun-Su Jang, Si-Woo Lee, Su-Hwa Lee, Chang-Gue Son

**Affiliations:** 1 Institute of Bioscience and Integrative Medicine, Department of Korean Medicine, Daejeon University, 62 Daehak-ro, Dong-gu, Daejeon, Republic of Korea; 2 Department of Health Service Management, Daejeon University, 96-3 Yongun-dong, Dong-gu, Daejeon, 300-716 Republic of Korea; 3 Division of Future Medicine, Korean Institute of Oriental Medicine, Yuseong-daero, 1672 Daejeon, Republic of Korea; 4 The Catholic University of Korea, Daejeon St. Mary Hospital, 64, Daeheung-ro, Jung-gu, Daejeon, Republic of Korea

**Keywords:** Chronic fatigue syndrome, CFS, ME/CFS, Prevalence, Systematic review, Meta-analysis

## Abstract

**Background:**

Chronic fatigue syndrome**/**myalgic encephalomyelitis (CFS/ME) has been emerging as a significant health issue worldwide. This study aimed to systemically assess the prevalence of CFS/ME in various aspects of analyses for precise assessment.

**Methods:**

We systematically searched prevalence of CFS/ME from public databases from 1980 to December 2018. Data were extracted according to 7 categories for analysis: study participants, gender and age of the participants, case definition, diagnostic method, publication year, and country of the study conducted. Prevalence data were collected and counted individually for studies adopted various case definitions. We analyzed and estimated prevalence rates in various angles: average prevalence, pooled prevalence and meta-analysis of all studies.

**Results:**

A total of 1291 articles were initially identified, and 45 articles (46 studies, 56 prevalence data) were selected for this study. Total 1085,976 participants were enrolled from community-based survey (540,901) and primary care sites (545,075). The total average prevalence was 1.40 ± 1.57%, pooled prevalence 0.39%, and meta-analysis 0.68% [95% CI 0.48–0.97]. The prevalence rates were varied by enrolled participants (gender, study participants, and population group), case definitions and diagnostic methods. For example, in the meta-analysis; women (1.36% [95% CI 0.48–0.97]) vs. men (0.86% [95% CI 0.48–0.97]), community-based samples (0.76% [95% CI 0.53–1.10]) vs. primary care sites (0.63% [95% CI 0.37–1.10]), adults ≥ 18 years (0.65% [95% CI 0.43–0.99]) vs. children and adolescents < 18 years (0.55% [95% CI 0.22–1.35]), CDC-1994 (0.89% [95% CI 0.60–1.33]) vs. Holmes (0.17% [95% CI 0.06–0.49]), and interviews (1.14% [95% CI 0.76–1.72]) vs. physician diagnosis (0.09% [95% CI 0.05–0.13]), respectively.

**Conclusions:**

This study comprehensively estimated the prevalence of CFS/ME; 0.89% according to the most commonly used case definition CDC-1994, with women approximately 1.5 to 2 folds higher than men in all categories. However, we observed the prevalence rates are widely varied particularly by case definitions and diagnostic methods. An objective diagnostic tool is urgently required for rigorous assessment of the prevalence of CFS/ME.

## Background

Chronic fatigue syndrome/myalgic encephalomyelitis (CFS/ME) is a debilitating illness that lacks a universally accepted case definition, cause, diagnosis, or treatment [1]. It is characterized by chronic fatigue lasting more than 6 months that is not alleviated by rest and is accompanied by complex and fluctuating symptoms of post exertion malaise (PEM), unrefreshing sleep, cognitive impairment, autonomic dysfunction, and/or pain in muscle or joint [2]. CFS/ME is known to be associated with poor health-related quality of life, worse than cancer, multiple sclerosis and stroke [3]. In fact, approximately 25 to 29% of CFS/ME patients were reported being house- or bed-bounded [4], also over half of the patients are unemployed [5], and only 19% work full-time [3].

Since this disorder was first recorded in 1934, numerous researchers have struggled to explore its biological etiologies, including viral infection and autoimmune dysregulation [6], neuroendocrine abnormality due to decreased hypothalamic–pituitary–adrenal (HPA) axis activity [7], and immune impairment caused by the abnormal production of cytokines [8]. Most recently, investigation of the interface between microglial activation and neuro-inflammation [9], the presence of widespread neuro-inflammation in the brains of CFS/ME patients [10], abnormal levels of serum TGF-*β*, a typical immune suppressive cytokine [11], and a nano-needle bioarray differentiating CFS/ME patients using blood samples [12], have provided new insights into the field. These studies may be promising; however, no therapeutics that can cure CFS/ME or objective diagnostic methods are available yet [13]. In addition, non-pharmacological therapies, such as cognitive behavior therapy (CBT) and graded exercise therapy (GET), and pharmacological trials, including immune modulator treatment, showed a lack of definitive efficacy for cure [13].

In worldwide statistics, approximately 1% of the population, 17 to 24 million people, suffer from this condition [14], which is likely to be as common as rheumatoid arthritis [15]. However, due to the lack of an objective diagnostic tool, an accurate estimation of prevalence has been challenging. Case definitions are the predominant tool for diagnosing CFS/ME at present. Since the development of the Centers for Disease Control and Prevention’s (CDC) 1988 definition, a number of case definitions have been developed, including the latest definition of systemic exertion intolerance disease (SEID) proposed by the Institute of Medicine in 2015 [2]. Discrepancies in prevalence have been demonstrated in several studies according to the case definition used; estimated prevalence of 2.6% with the CDC-1994 vs. 1.2% with the Holmes definition [16], and 0.42% with the CDC-1994 but increased by 2.8 times with the SEID definition [17].

Accurate prevalence rate and defining factor-related prevalence characteristics are essential for exploring the pathophysiological basis of any disease [18]. To date, several studies have estimated the overall prevalence rate of CFS/ME [19, 20]; however, they lacked in multi-analysis. This study aimed to provide comprehensive data on CFS/ME prevalence from multiple aspects, which will be helpful in future studies of CFS/ME.

## Methods

### Study design

To investigate the prevalence of CFS/ME and explore its features, a primary population-based study from public databases was systematically reviewed and analyzed. The extracted and collected data were combined for a meta-analysis to analyze the consistency of the prevalence. This systematic review has been registered in the PROSPERO database (CRD42019141250).

### Data sources and eligibility criteria

We searched research papers published from 1980 to December 2018, as the known first case definition published was in 1986 [21]. The data were collected from the databases PubMed, the Cochrane Library, EBSCOhost (CINAHL, Medline), Google Scholar, and hand-searched for relevant references. The search concepts were chronic fatigue syndrome, myalgic encephalomyelitis, prevalence, and clinical study, and the search keyword was “(Chronic fatigue syndrome [MeSH term]) AND Prevalence”.

**Fig. 1:**
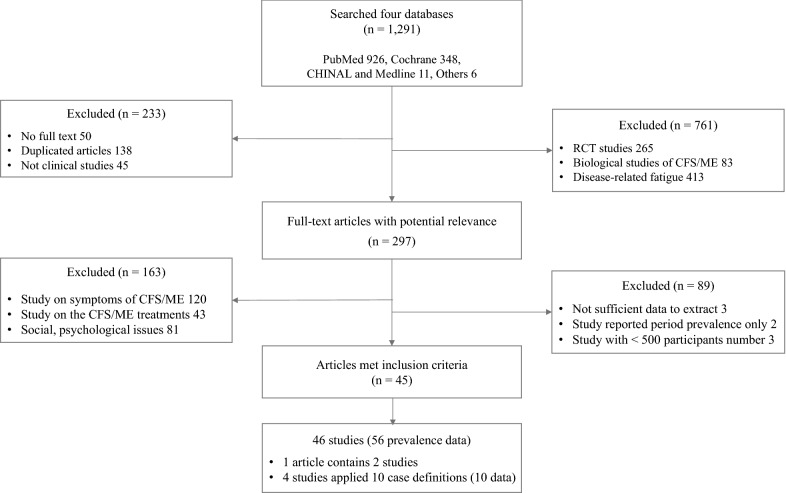
PRISMA flowchart. Study flowchart of the articles included in the analysis according to the Preferred Reporting Items for Systematic Reviews and Meta-Analyses. *CFS* chronic fatigue syndrome, *ME* myalgic encephalitis

Papers were screened using the following inclusion criteria: (a) prevalence study of chronic fatigue syndrome and/or myalgic encephalomyelitis, (b) clinical study, and (c) population-based study. The initial assessment was made by considering the inclusion criteria and reading the title and abstract. Articles that met the criteria were thoroughly read in full and screened according to the exclusion criteria. The exclusion criteria were as follows: (a) nonclinical-based studies, (b) studies on clinical features or symptoms of CFS/ME, (c) randomized controlled studies, (d) studies focusing on biological aspects of CFS/ME, (e) studies on psychological/psychiatric issues associated with CFS/ME, (f) studies on treatment or therapeutic aspects (e.g., the use of supplements) of CFS/ME, and (g) studies with fewer than 500 participants (Fig. 1).

### Review process and data extraction

Two authors initially searched and selected the eligible articles according to the above inclusion and exclusion criteria. From the selected articles, data pertaining to prevalence, the number of participants and CFS/ME patients, gender of the participants, population group, study participants, publication year, country, case definition, and diagnostic method were extracted. In particular, case definitions were extracted and treated as individual prevalence data for studies that applied multiple case definitions. The selection of articles, the clarity of the extracted data, and the final decision was based upon the consensus of our research team.

### Data coding and synthesis

The data from each article were subgrouped as follows: the age of the participants; publication year (1990–2000, 2001–2010, 2010–2018); study participants (community, primary care); population group (general population, children and adolescents, specific population); country (Western, Asian, others); 8 case definitions: CDC-1994 (Centers for Disease Control and Prevention) [22], Holmes [23], Oxford [24], Australian [25], and others, including ECD (epidemiological case definition) [26], CCC (Canadian Consensus Criteria) [27], PVES (post viral exhaustion syndrome) [28], NICE guidelines (National Institute for Health and Care Excellence) [29], and diagnostic methods (interviews with/without medical test, physician diagnosis, medical record). These data were coded as categorical variables and synthesized in a coding book developed by our research team. The number of participants and CFS/ME patients from the selected articles were arranged to compare community samples vs. primary care samples and males vs. females to estimate the number of populations and the prevalence. The codes and details of the 45 selected articles are provided in the supplementary data (Additional file 1).

### Statistical analysis

A meta-analysis using the random effects model by the *R* program was conducted to account for the heterogeneity of the data: prevalence with subgroup analysis was applied. Heterogeneity indicates dissimilarity in the individual study results. *I*^2^ quantifies the effect of heterogeneity; that is, the proportion of interstudy variability [30, 31].

## Results

### Characteristics of the included studies

From four major public databases, a total of 1291 articles were initially identified, and 45 articles finally met the inclusion criteria for this study. The first study of selected articles was reported in 1990 [25], and the latest one was in 2018 [32]. Forty-six studies (one article contained two studies) were conducted in 13 countries (41 prospectively and 5 retrospectively). Four studies applied multiple case definitions (2 studies applied 4 case definitions, 2 studies applied 3 case definitions), providing a total of 56 prevalence data (Fig. 1). The total number of participants was 1085,976 from 30 community-based studies (33 data, n = 540,901) and 16 primary care studies (23 data, n = 545,075). Thirty-four studies (44 data) focused on the general population (n = 956,526, age ≥ 18 years), 7 focused on children and adolescents (n = 117,307, < 18 years), and 5 focused on a particular population (n = 12,143; nurses, 2; company employees, 1; livestock employees, 1; Gulf War veterans, 1). The average number of participants in each study was 23,608 ± 48,092 (± SD). Of the 46 studies, 21 studies (24 data) reported gender-related information (males, n = 62,070; females, n = 68,772) (Table 1).

**Table 1:** Characteristics of studies on the prevalence of CFS/ME (± SD)

Group	Community		Primary care		Total	
Number of articles included (%)	30 (67)		15 (33)		45 (100)	
Number of studies included^a^(%)	30 (65)		16 (35)		46 (100)	
Prospective	25 (54)		16 (35)		41 (89)	
Retrospective	4 (9)		1 (2)		5 (11)	
Number of prevalence data^b^ (%)	33 (59)		23 (41)		56 (100)	
Total number of participants	540,901		545,075		1085,976	
Mean *N*. of participants	18,030 ± 38,094		34,067 ± 61,325		23,608 ± 48,092	
Total number of known sex^c^	120,765		10,077		130,842	
Male	58,752		3318		62,070	
Female	62,013		6759		68,772	
M:F ratio	48:52		25:75		45:55	
Mean age of participants^d^	41.1 ± 11.3		38.0 ± 6.5		40.0 ± 9.9	
Male	41.1 ± 11.3		38.0 ± 6.8		39.9 ± 10.0	
Female	41.2 ± 11.4		38.1 ± 6.1		40.0 ± 9.8	
*N*. of (studies)/prevalence data by subgroup (*N*. of participants)						
Publication year						
1990–2000	8	(177,201)	15	(318,391)	(18)/23	(495,592)
2001–2010	19	(86,512)	4	(9895)	(20)/23	(96,407)
2011–2018	6	(277,188)	4	(216,789)	(8)/10	(493,977)
Population group						
General population (≥ 18 years)	24	(489,961)	20	(466,565)	(34)/44	(956,526)
Children/adolescents (< 18 years)	6	(43,671)	1	(73,636)	(7)/7	(117,307)
Specific population^e^	3	(7269)	2	(4874)	(5)/5	(12,143)
Case definition (8 case definitions)	23	(339,192)	11	(298,739)	34	(637,931)
CDC-1994 [22]	4	(20,037)	4	(27,454)	8	(47,491)
Holmes [23]	2	(116,520)	2	(2980)	4	(119,500)
Australian [25]	2	(3215)	2	(2980)	4	(6195)
Oxford [24]	1	(10,396)	3	(505,299)	4	(515,695)
CCC [27] ECD [26] PVES [28] NICE [29] ^f^ N/A^g^	1	(59,101)	1	(1874)	2	(60,975)
Country (13 countries)						
Western	27	(516,617)	17	(473,009)	(34)/44	(989,626)
Asian	5	(23,197)	5	(70,192)	(10)/10	(93,389)
Others^h^	1	(1087)	1	(1874)	(2)/2	(2961)
Diagnostic method						
Interview (medical test −)	19	(111,943)	3	(68,848)	(19)/22	(180,791)
Interview (medical test +)	9	(57,339)	14	(17,445)	(18)/23	(74,784)
Physician diagnosis	1	(114,000)	5	(435,782)	(4)/6	(549,782)
Medical records	4	(257,619)	1	(23,000)	(5)/5	(280,619)

^a^The number of studies is larger (n = 46) than the number of article as one article included two studies

^b^Some articles included multiple applications of case definitions; thus, the number is larger than the total number of studies

^c^Twenty-one studies (24 prevalence data points) included information about participant sex

^d^The mean age of the participants whose sex was known (12 studies) was estimated using either the reported mean age for each sex or the mean age for both. Children and adolescents were excluded

^e^Specific groups included nurses, Gulf War veterans, livestock workers, company employees, etc

^f^CCC, Canadian Consensus Criteria; ECD, epidemiological case definition; PVES, post viral exhaustion syndrome; and NICE, National Institute for Health and Care Excellence guideline 2007

^g^Two N/A cases had no verification of case definition or defined criteria

^h^Other countries included India and Nigeria

A total of 8 case definitions were adopted in 46 studies (56 data); the most frequently used definitions were CDC-1994 (34 data) and Holmes (8 data). The majority of the studies (37 studies/45 data) used interview-based diagnoses with/without medical tests, and the remaining studies (9 studies/11 data) were based on a physicians’ diagnosis/determination and medical record reviews. The average age of all participants except children and adolescents, based on the mean of the means/median ages reported in the articles, was 40.0 ± 9.9 years (Table 1).

### Overview of CFS/ME prevalence

**Fig. 2:**
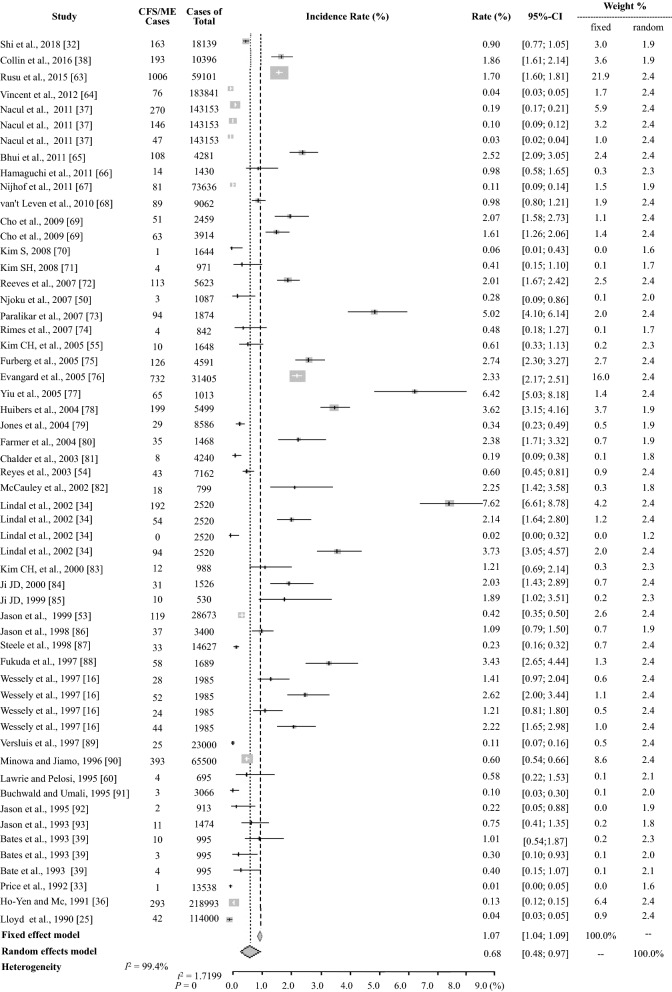
Meta-analysis of the total CFS/ME prevalance. *CFS* chronic fatigue syndrome. *ME* myalgic encephalitis

**Fig. 3:**
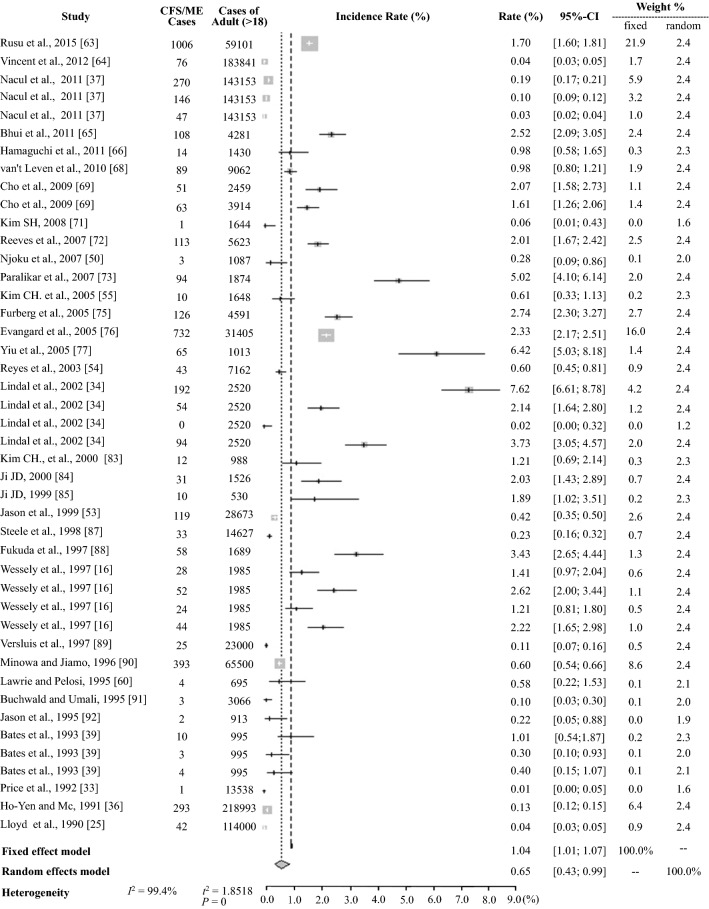
Meta-analysis of the CFS/ME prevalence in adults (≥ 18 years). *CFS* chronic fatigue syndrome, *ME* myalgic encephalitis

The average prevalence of CFS/ME based on the 56 prevalence data reported was 1.40 ± 1.57% (95% CI: 0.98–1.82), and the pooled prevalence was 0.39% (5370 CFS/ME patients of 1387,787 participants) (Table 2). The meta-analysis yielded an estimate of 0.68% (95% CI: 0.48–0.97) with high heterogeneity *I*^2^ = 99.4% (Table 3, Fig. 2). For the general population (excluding data for children and adolescents and specific populations), the average prevalence was 1.45 ± 1.68% (95% CI: 1.01–1.89) and 0.38% (4724 CFS/ME patients of 1258,337 participants) and 0.65% (95% CI: 0.43–0.99) in the meta-analysis based on 44 prevalence data (Tables 2 and 3, and Fig. 3).

**Table 2:** Prevalence of CFS/ME by subgroup (± SD)

Group^a^	Male (%)	Female (%)	Total (%, M/F)	Total (%)
	(21 studies/24 data)			(46 studies/56 data)
Average prevalence of all studies	1.11 ± 1.05	2.24 ± 2.59	1.67 ± 2.06	1.40 ± 1.57
Pooled prevalence of all studies (Total *N*. of CFS/*N*. of participants^b^)	0.74 (451/61,069)	1.92 (1308/68,124)	1.37 (1778/129,780)	0.39 (5370/1387,787)
Pooled prevalence of the general population (*N*. of adult CFS/*N*. of adult participants)	0.75 (451/60,432)	1.92 (1304/67,790)	1.38 (1774/128,809)	0.38 (4724/1258,337)
Mean age of CFS patients (12 studies)	39.3 ± 7.8	39.1 ± 7.6	40.4 ± 7.7	40.4 ± 7.7
Prevalence by subgroup (*N*. of studies that reported sex)				
Study participants				
Community (16)	1.03 ± 1.13	2.31 ± 2.88	1.67 ± 2.28	1.56 ± 1.80
Pooled prevalence (*N*. of CFS/*N*. of participants^b^)	0.70 (404/57,751)	1.94 (1190/61,365)	1.34 (1594/119,116)	0.73 (4014/548,461)
Primary care (5)	1.39 ± 0.56	1.96 ± 0.74	1.68 ± 0.72	1.16 ± 1.13
Pooled prevalence (*N*. of CFS/*N*. of participants^b^)	1.42 (47/3318)	1.75 (118/6759)	1.64 (165/10,077)	0.21 (1739/839,326)
Publication year				
1990–2000 (9)	0.62 ± 0.58	1.26 ± 1.42	0.94 ± 1.13	0.96 ± 0.91
2001–2010 (10)	1.35 ± 1.18	2.99 ± 3.11	2.17 ± 2.49	2.08 ± 2.01
2011–2018 (2)	1.75 ± 0.83	1.78 ± 0.71	1.76 ± 0.77	0.84 ± 0.86
Population group				
General population (≥ 18 years) (19)	1.39 ± 1.05	2.83 ± 2.61	2.11 ± 2.07	1.45 ± 1.68
Children/adolescents (< 18 years) (0)	–	–	–	0.89 ± 0.82
Specific population^c^(1)	0.12	0.06	0.09 ± 0.03	1.62 ± 1.17
Case definition (8 case definitions)				
CDC-1994 [22] (16)	1.24 ± 1.04	2.61 ± 2.75	1.93 ± 2.19	1.46 ± 1.34
Holmes [23] (4)	0.07 ± 0.05	0.14 ± 0.15	0.11 ± 0.12	0.34 ± 0.40
Australian [25] (1)	2.65	5.23	3.94 ± 1.29	2.52 ± 2.99
Oxford [24] (2)	1.23 ± 0.64	1.76 ± 1.22	1.51 ± 1.00	1.73 ± 1.35
CCC [27] ECD [26] PVES [28] NICE [29]^d^(0)	–	–	–	0.53 ± 0.77
Country (13 countries)				
Western (12)	1.14 ± 0.97	2.40 ± 2.86	1.77 ± 2.22	1.32 ± 1.45
Asian (8)	1.23 ± 2.92	2.06 ± 1.85	1.65 ± 1.64	1.51 ± 1.74
Others^e^(1)	0.11	0.50	0.31 ± 0.20	2.65 ± 2.37
Diagnostic method				
Interview (medical test -) (9)	1.70 ± 1.21	4.32 ± 3.24	3.01 ± 2.63	2.03 ± 2.13
Interview (medical test +) (10)	0.86 ± 0.70	1.23 ± 0.93	1.05 ± 0.84	1.17 ± 0.77
Physician diagnosis (0)	–	–	–	0.10 ± 0.05
Medical records (1)	2.57	2.49	2.53 ± 0.06	1.25 ± 1.00

^a^The prevalence by sex was estimated from studies that reported both the number of participants and the number with CFS

^b^Participant number was applied to individual prevalence data for the studies with multiple case definitions

^c^Specific groups included nurses, Gulf War veterans, livestock workers, company employees, etc

^d^CCC, Canadian Consensus Criteria; ECD, epidemiological case definition; PVES, post viral exhaustion syndrome; and NICE, National Institute for Health and Care Excellence guideline 2007; two studies with no verification of case definition and defined criteria were excluded

^e^Other countries included India and Nigeria

**Table 3:** Meta-analysis results for the prevalence of CFS/ME

Group	*N*. of data^a^	Random effects model (%)		Heterogeneity (*P *< 0.01, *P *= 0%)		
		Prevalence	95% CI	*Q*	*T*^*2*^	*I*^*2*^ (%)
Total	56	0.68	[0.48; 0.97]	8602.90	1.7199	99.4
Sex						
Male	24	0.86	[0.58; 1.27]	279.89	0.6666	91.8
Female	24	1.36	[0.91; 2.02]	822.64	0.8003	97.2
Total	48	1.04	[0.76; 1.41]	1481.76	0.9471	96.8
Study participants						
Community	33	0.76	[0.53; 1.10]	3286.47	1.0363	99.0
Primary care	23	0.63	[0.37; 1.10]	2732.10	1.7745	99.2
Population group						
General population (≥ 18 years)						
Male	23	0.89	[0.60; 1.32]	276.23	0.6607	92.0
Female	23	1.36	[0.91; 2.04]	818.30	0.8009	97.3
Total	44	0.65	[0.43; 0.99]	7717.65	1.8518	99.4
Children/adolescents (< 18 years)	7	0.55	[0.22; 1.35]	538.13	1.4319	98.9
Specific population	5	1.31	[0.61; 2.78]	80.54	0.6657	95.0
Case definition						
CDC-1994 [22]	34	0.89	[0.60; 1.33]	3871.64	1.3691	99.1
Holmes [23]	8	0.17	[0.06; 0.49]	101.72	1.8890	93.1
Australian [25]	4	0.79	[0.05; 12.55]	1002.43	7.8860	99.7
Oxford [24]	4	1.41	[0.68; 2.93]	35.17	0.4468	91.5
CCC [27] ECD [26] PVES [28] NICE [29]^b^	4	0.17	[0.04; 0.83]	1200.67	2.5864	99.8
Diagnostic method						
Interview (medical test −)	22	1.14	[0.76; 1.72]	1675.91	0.8269	98.7
Interview(medical test +)	23	0.95	[0.69; 1.31]	365.72	0.5208	94.0
Physician diagnosis	6	0.09	[0.05; 0.13]	200.49	0.2952	97.5
Medical records	5	0.52	[0.16; 1.71]	1197.28	1.8360	99.7

Refer to supplementary Additional file: Figs. 1, 2, 3, 4, 5, 6, 7

^a^Total number of prevalence data points

^b^CCC, Canadian Consensus Criteria; ECD, epidemiological case definition; PVES, post viral exhaustion syndrome; and NICE, National Institute for Health and Care Excellence guideline 2007; two studies with no verification of case definition and defined criteria were excluded

**Fig. 4:**
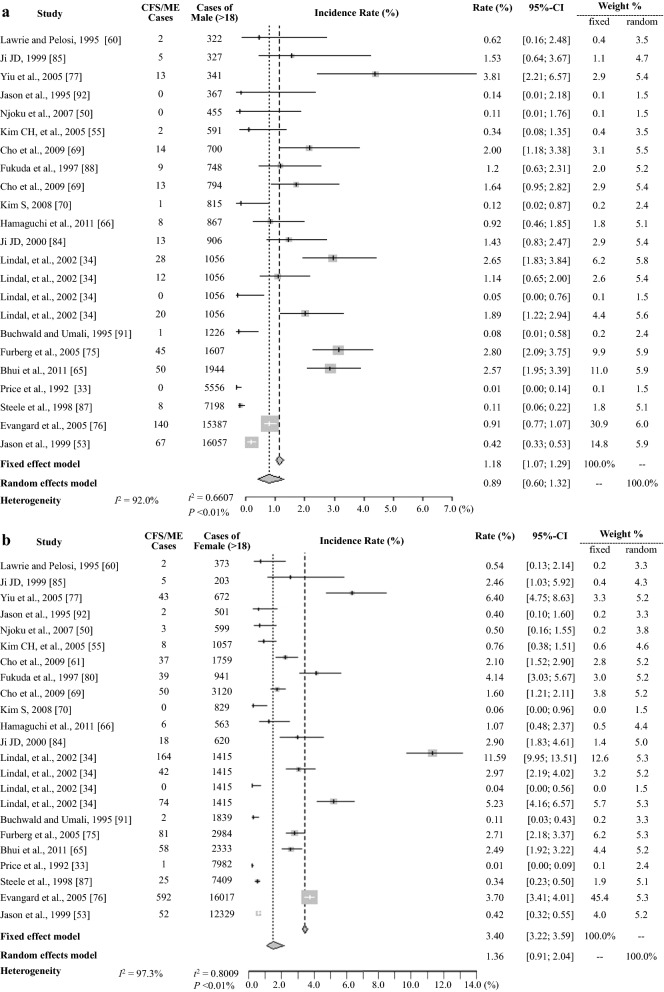
Meta-analysis of the CFS/ME prevalence in males **a**, females **b**. *CFS* chronic fatigue syndrome, *ME* myalgic encephalitis

Regarding gender-related differences, 24 data (from 21 studies that included information about gender) indicated an approximately 2.0-fold preponderance of females of 2.24 ± 2.59% vs. 1.11 ± 1.05% for the total population and 2.83 vs. 1.39% for the general population; the prevalence was 1.5-fold higher according to the meta-analysis, 1.36 vs. 0.89% (Table 2 and 3, Fig. 4).

### Prevalence by study participants

When we analyzed the CFS/ME prevalence according to study participants (community-based sample vs. primary care sample), the 30 community studies (33 data) showed a prevalence of 1.56 ± 1.80% (95% CI: 1.08–2.04), and the 16 primary care studies (23 data) showed a prevalence of 1.16 ± 1.13% (95% CI: 0.86–1.46). A higher prevalence in community studies was also observed in the pooled prevalence results of 0.73% (4014 CFS/ME patients out of 548,461 participants) vs. 0.21% (1739 out of 839,326) and in the meta-analysis findings of 0.76% (95% CI: 0.53–1.10) vs. 0.63% (95% CI: 0.37–1.10), respectively (Table 2, 3, and Additional file 2). From the results of 21 studies (24 data) with known gender-related data, the prevalence of female preponderates in both community (2.31% vs. 1.03%) and primary care settings (1.96 vs. 1.39%) (Table 2).

### Prevalence by study population group

The study population was divided into 3 groups: general population (n = 956,526, ≥ 18 years), children and adolescents (n = 117,307, < 18 years), and specific populations (n = 12,143, e.g., nurses, Gulf War veterans) (Table 1). The prevalence in the general population (1.45%) and in specific populations (1.62%) were higher than that in children and adolescents (0.89%) (Table 2). The meta-analysis indicated prevalence of 0.65% (95% CI: 0.43–0.99) in the general population, 1.31% (95% CI: 0.61–2.78) in specific populations and 0.55% (95% CI: 0.22–1.35) in children and adolescents (Table 3, Fig. 3, and Additional file 3). In the general population, a female predominance was shown in both the averaged (2.83 ± 2.61 vs. 1.39 ± 1.05%), and the meta-analysis (1.36%, 95% CI: 0.91–2.04 vs. 0.89, 95% CI: 0.60–1.32) (Table 2, 3 and Fig. 4).

### Prevalence by case definitions

Eight case definitions [22–29] were adopted for 44 studies (54 data); 2 studies used unknown case definitions. The total prevalence was notably different according to case definition:, the prevalence was the highest with Australian (2.52 ± 2.99%), then in descending order, Oxford (1.73 ± 1.35%), CDC-1994 (1.46 ± 1.34%) and Holmes definitions (0.34 ± 0.40%), but in the meta-analysis, the orders changed to Oxford (1.41%, 95% CI: 0.68–2.93), CDC-1994 (0.89%, 95% CI: 0.60–1.33), Australian (0.79%, 95% CI: 0.05–12.55) and Holmes (0.17%, 95% CI: 0.06–0.49) (Table 2, 3 and Additional file 4, 5).

### Prevalence by diagnostic method

In this study, the diagnostic methods could be classified into four groups, and the prevalence data differed significantly among them (*P* < 0.05), as follows: interview without a medical test (survey and/or questionnaire, 19 studies, averaged prevalence 2.03 ± 2.13%), interview with a medical test (18 studies, 1.17 ± 0.77%), review of medical records (5 studies, 1.25 ± 1.00%) and physician diagnosis (4 studies, 0.10 ± 0.05%) (Table 1, 2).

The meta-analysis also showed different prevalence, as follows: interviews without medical tests (1.14%, 95% CI: 0.76–1.72), interviews with medical tests (0.95% 95% CI: 0.69–1.31), medical record review (0.52% 95% CI: 0.16–1.71), and physician diagnoses (0.09%, 95% CI: 0.05–0.13) (Table 3, and Additional file 6, 7).

### Prevalence by country and publication year

The majority of the studies were conducted in Western countries (34 studies/44 prevalence data from 8 countries, 989,626 participants), followed by Asian countries (10 studies/10 data, 3 countries, 93,389), and others (2 studies/2 data, 2 countries, 2961). The total prevalence reported for Western and Asian populations were comparable (1.32 ± 1.45% vs. 1.51 ± 1.74%) (Tables 1, 2 and Additional file 1). The majority (38 of 46) of the studies were published between 1990 and 2010. Of those studies, 10 (of 18) studies were for primary care population conducted in 1990s, whereas 16 (of 20) for community in 2000s. More studies on community-based were conducted in 2000s than 1990s (Table 1). The total prevalence in 2000s (2.08 ± 2.01) was approximately twofold higher than 1990s (0.96 ± 0.91) (Table 2).

## Discussion

This systematic review and meta-analysis aimed to provide a reviewed estimate of the prevalence of CFS/ME worldwide. We combined the 56 data from 46 studies conducted in 13 countries since prevalence study was first published in 1990 for the Australian general population [25]. The prevalence of CFS/ME varies widely, from 0.01 [33] to 7.62% [34], as indicated by the high heterogeneity in the meta-analysis, *I*^2^ = 99.4% (Table 3 and Additional file 1). We considered the matter from various angles to investigate the inconsistency of the prevalence data. Thus, we synthesized the prevalence data, estimated the average, the pooled prevalence based on the number of participants and CFS/ME patients, and assessed the prevalence with heterogeneity by using meta-analysis according to the following subgroups: gender, study participants, population group, case definition, diagnostic method, and country.

**Fig. 5:**
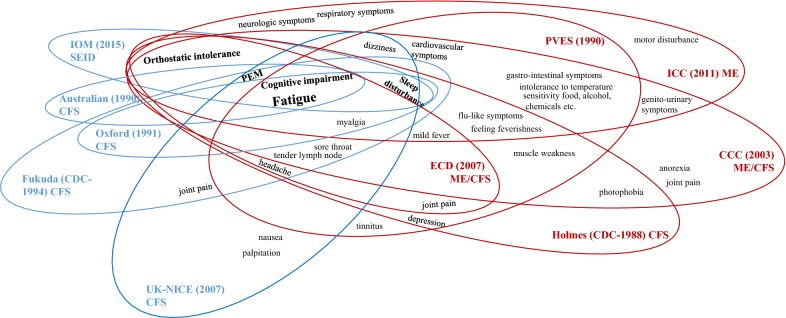
Key symptoms of CFS/ME by case definitions. *CFS* chronic fatigue syndrome, *ME* myalgic encephalitis, *PEM* postexertion malaise, *IOM* Institute of Medicine, *SEID* systemic exertion intolerance disease, *NICE* National Institute for Health and Care Excellence, *PVES* Post viral exhaustion syndrome. Holmes. *ECD* epidemiology case definition, *CCC* Canadian Consensus Criteria, *ICC* International Consensus Criteria

The terms and case definitions for CFS and ME have been reformulated according to perceptions of the disorder and study groups throughout the history of the disorder (Fig. 5). Briefly, this condition was thought to be a new type of poliomyelitis in the 1930s and was then perceived as hysteria caused by psychological issues in the 1970s and early 1980s. Since Ramsay M. defined the first diagnostic criteria for ME in 1986, characterizing it as a unique form of muscle fatigability triggered by a virus [2], various terminologies and case definitions have been proposed. In 1988, the CDC first proposed the new term CFS (Holmes criteria) instead of “Chronic Epstein-Barr virus syndrome” to more accurately describe the symptom complex requiring 2 major with 8 of 11 minor symptoms, and to emphasize recurrent debilitating fatigue [23]. The term was revised by Fukuda K. in 1994 (Fukuda criteria) [22]. In 2003, CFS/ME, an umbrella term that covers both ME and CFS symptom criteria, was used in the CCC definition [27], while the ICC reformulated the definition and readopted the term ME in 2011 [35], and a new term, SEID (Systematic exertion intolerance disease), and its criteria were suggested by the IOM in 2015 [2].

As expected, the prevalence rates of CFS/ME differed according to the case definitions; there was an approximately sevenfold difference in the averaged prevalence of 0.34% based on the Holmes definition vs. 2.52% based on the Australian definition (Table 2). In general, the definitions could be categorized into two groups: the CDC-1994 [22], Australian [25], Oxford [24], IOM-SEID [2] and NICE definitions [29] vs. the Ramsay [21], Holmes [23], ICC 35], CCC [27], PVES [28], and ECD definitions [26]. These two groups overlap in requiring the symptom of cognitive impairment and share the general physical and neurologic symptoms; however, they differ in their inclusion or exclusion of more immune-, neuroendocrine-, and/or autonomic-related symptoms (Fig. 5). Accordingly, in our study, the prevalence determined with the CDC-1994, Australian, Oxford, NICE definitions were higher (range 1.46–2.52%) than those determined using the definitions CCC, ECD, PVES, and Holmes definitions (range 0.03–0.34%) [36–38] (Table 2, Fig. 5). Four studies that independently applied multiple case definitions to the same population all reported considerably higher prevalence based on the CDC-1994, Australian, and Oxford definitions than on the Holmes definition [16, 34, 37, 39].

The majority of the prevalence data (34 of total 56 data) were based on the CDC-1994 definition in our study, which found a mean prevalence of 1.46% and a meta-analysis result of 0.89% (Tables 2 and 3). Our mean prevalence result (1.46%) is comparable to the results of previous review studies of CFS/ME prevalence that adopted the CDC-1994 definition [20, 40]. The pathophysiology of CFS/ME is still unclear; thus, the definition of this disorder is not yet conclusive. The CDC-1994 definition is criticized of the polythetic method that can select some individuals without the core symptoms of CFS [41, 42]. The most recent definition, SEID, is also said to be a problematic due to the possibility of including psychiatric illness [43]. This suggests a need for a rigorous diagnostic procedure with clear cut-off points and reasons for exclusions that anticipates the presence of subtypes of CFS/ME patients [20, 44], and the objective diagnostic parameters [45].

As expected, prevalence can also vary by study design. Among the 4 categories of diagnostic methods, the prevalence rate based on physician diagnosis was the lowest (0.10%). The questionnaire-based interview without a medical test yielded the highest prevalence (2.03%), while the addition of a medical test reduced the prevalence by approximately half a percentage point (1.17%) (Table 2). A similar pattern was observed in the meta-analysis (1.14% vs. 0.95%, Table 3, Additional file 6) and was also described in Johnston’s review study [19]. In the clinical field, the final diagnosis of certain diseases is made by a physician on the basis of medical tests; accordingly, it is anticipated that only the questionnaire-derived CFS/ME prevalence is likely to be overestimated. On the other hand, there are concerns that physicians tended to deny the diagnosis or to not believe in CFS as a disease [46]. In addition, the complexity and rarity of the condition should be considered in terms of the diagnosis and management of CFS in general practice [47].

It is well-known genetic background and living environment are important factors in the development or progression of diseases [48]. CFS/ME was once considered a disease of the middle to upper classes that was mostly prevalent in the Caucasian population [49], although other studies have suggested that members of minority groups and lower economic classes are more prone to CFS/ME due to psychosocial and environmental risk factors such as lack of adequate nutrition, limited access to healthcare, and work-related stressors [16, 50–52]. In this respect, it is of interest that some studies from different countries showed similar prevalence rates in similar settings; i.e., when the CDC-1994 was used with a medical test for a community-based general population, similar results were found for Nigeria (0.28% for CFS, or 0.68% of CFS-like), the U.S. (0.60% and 0.42%), and Korea (0.61%) [50, 53–55] (Table 2). Additionally, the prevalence in specific populations, such as nurses and Gulf War veterans, seems to be slightly higher (1.62%) than that in the general population (1.45%) (Table 2); however, as others have argued, this difference could result from methodological inconsistencies [18].

In our results, women had CFS/ME prevalence approximately 1.5- to 2 fold higher than that of men, and this finding was consistent in all subgroups (Table 1, 2). This gender difference in CFS/ME prevalence could be related to biological factors, primarily gender hormones and/or immunologic responses to environmental exposures [56, 57]. Some review studies reported a gender difference starting at puberty (approximately 13 years of age) in anticipation of hormonal or biochemical responses [48, 58, 59]. Our results showed a 0.89% (0.55% in the meta-analysis) prevalence in children and adolescents based on data from 6 studies (Tables 2 and 3). A Norwegian population-based study showed a 3.2-fold female predominance, and, interestingly, two age peaks for prevalent features in both gender: ages 10–19 and 30–39 years [59]. A further epidemiological study of biological changes according to those age peaks may support a rational for the gender differences. Furthermore, the greatest gender difference (females 1.94% vs. males 0.70%) was shown in the pooled prevalence for the community population (Table 2). As described above, data for the community-based studies were mainly conducted by using interview-based methods (Table 1), and additionally, women are known to be more likely to report their complaints [60].

This study provides an updated review on the prevalence of CFS/ME but does not assess the accuracy of diagnosis. The limitations are the high heterogeneity of diagnostic tools and methods used; the lack of data based on some case definitions, such as ICC, CCC and SEID; the small number of studies in some subgroup analyses; and limited information on gender and age. Despite these limitations, we found that there was some possibility of under- or overestimation of the prevalence, particularly depending on the case definitions adopted. We observed a high heterogeneity in the reported prevalence; as estimated, the ranges for three extraction methodologies were 1.40% (95% CI: 0.98–1.82) for the averaged prevalence, 0.39% (95% CI: 0.00–0.81) for the pooled prevalence and 0.68% (95% CI: 0.48–0.97) for the meta-analysis. Recently, one study reported a 0.67% CFS or ME prevalence and a 0.12% ME prevalence using large medical claims data with ICD (International Classification of Disease) codes [61]. Those results concur with our CFS/ME prevalence findings of 0.68% for the entire dataset and 0.09 and 0.12% in the meta-analysis based on physician diagnosis and the Holmes definition, respectively. Thus, case definition and diagnostic methods are the factors with the greatest influence on the results, with data ranges that vary by approximately 5- to tenfold. Following our study results, in addition to a proposal for a new diagnostic code [61, 62], a pattern recognition methods to subdivide CFS patients according to symptom clusters (e.g., specific phenotype features) with the adaption of objective measurement (e.g., two cardiopulmonary exercise tests, CPETs) were suggested for more empiric definition of the condition [44].

## Conclusions

Taken together, our findings illustrated the prevalence of CFS/ME, providing comprehensive information that can serve as an essential reference for further studies of CFS/ME. The overall estimated prevalence was 0.89% when based on the CDC-1994 definition and 1.14% when diagnosed via interview, and there was an approximately 1.5-fold predominance of women; however, the prevalence rates varied according to the case definitions and diagnostic methods used by as much as tenfold.

## Supplementary information

**Additional file 1:** Extracted raw data from the included studies.

**Additional file 2:** Meta-analysis of CFS/ME prevalence in community (A) and primary care (B) settings.

**Additional file 3:** Meta-analysis of the CFS/ME prevalence for children and adolescents (A) and specific populations (B).

**Additional file 4:** Meta-analysis of CFS/ME prevalence based on the CDC-1994 case definition.

**Additional file 5:** Meta-analysis of CFS/ME prevalence based on the Holmes (A), Australian (B), Oxford (C), and other (D) definitions.

**Additional file 6:** Meta-analysis of CFS/ME prevalence by interview (A) and interview with medical test (B).

**Additional file 7:** Meta-analysis of prevalence studies with diagnosis by physician determination (A) and review of medical records (B).

## Data Availability

All data analyzed during this study are available in the public domain.
