# Supplementary material for: Systematic review and meta-analysis of the prevalence of chronic fatigue syndrome/myalgic encephalomyelitis (CFS/ME)
Source: J Transl Med. 2020 Feb 24;18:100. doi: 10.1186/s12967-020-02269-0 (PMC7038594; doi:10.1186/s12967-020-02269-0)
Supplement: Supplementary file 1 — Additional file 1: Extracted raw data from the included studies. [file 12967_2020_2269_MOESM1_ESM.pptx]

## Slide 1
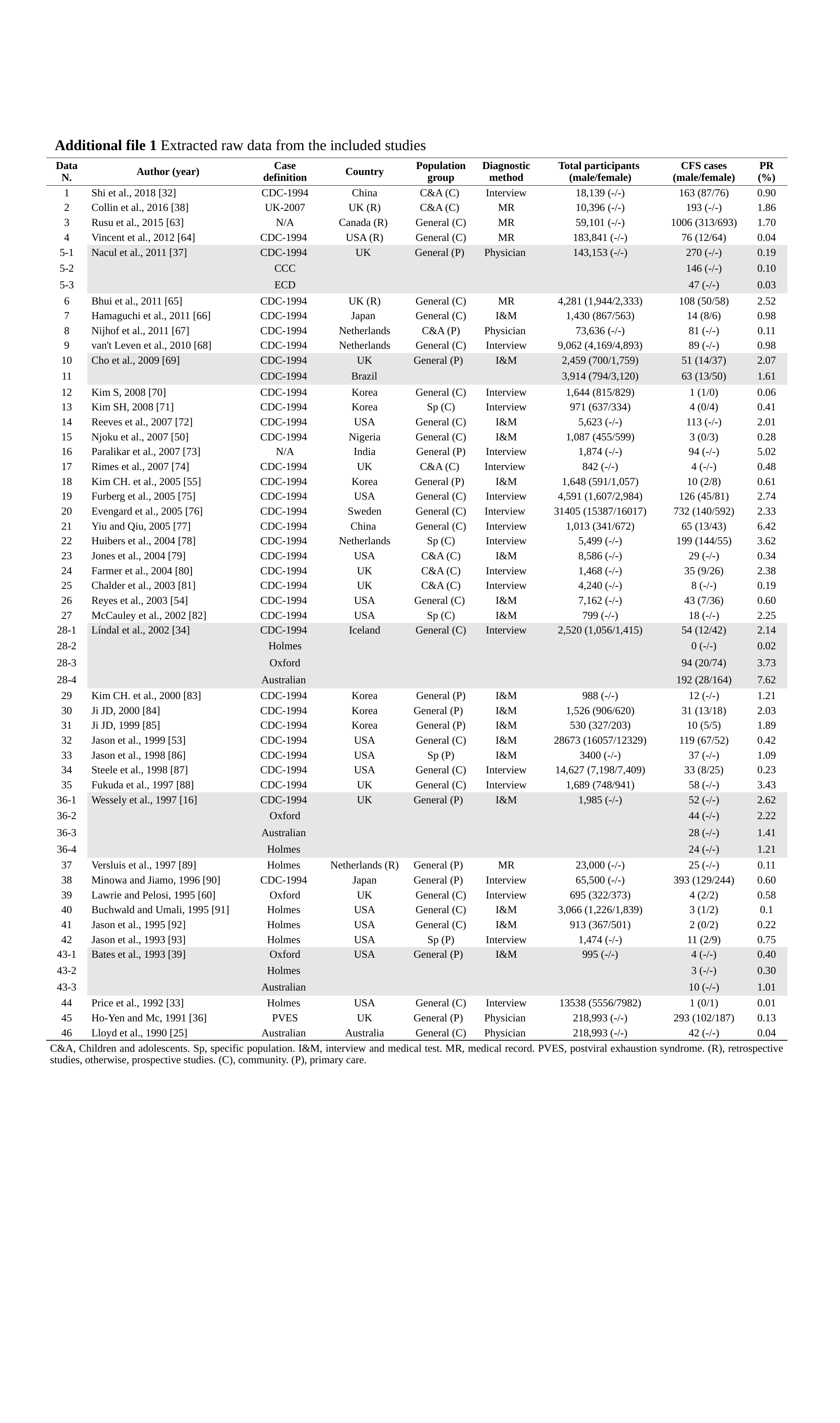

Additional file 1 Extracted raw data from the included studies
| Data N. | Author (year) | Case definition | Country | Population group | Diagnostic method | Total participants (male/female) | CFS cases (male/female) | PR (%) |
| --- | --- | --- | --- | --- | --- | --- | --- | --- |
| 1 | Shi et al., 2018 [32] | CDC-1994 | China | C&A (C) | Interview | 18,139 (-/-) | 163 (87/76) | 0.90 |
| 2 | Collin et al., 2016 [38] | UK-2007 | UK (R) | C&A (C) | MR | 10,396 (-/-) | 193 (-/-) | 1.86 |
| 3 | Rusu et al., 2015 [63] | N/A | Canada (R) | General (C) | MR | 59,101 (-/-) | 1006 (313/693) | 1.70 |
| 4 | Vincent et al., 2012 [64] | CDC-1994 | USA (R) | General (C) | MR | 183,841 (-/-) | 76 (12/64) | 0.04 |
| 5-1 | Nacul et al., 2011 [37] | CDC-1994 | UK | General (P) | Physician | 143,153 (-/-) | 270 (-/-) | 0.19 |
| 5-2 | | CCC | | | | | 146 (-/-) | 0.10 |
| 5-3 | | ECD | | | | | 47 (-/-) | 0.03 |
| 6 | Bhui et al., 2011 [65] | CDC-1994 | UK (R) | General (C) | MR | 4,281 (1,944/2,333) | 108 (50/58) | 2.52 |
| 7 | Hamaguchi et al., 2011 [66] | CDC-1994 | Japan | General (C) | I&M | 1,430 (867/563) | 14 (8/6) | 0.98 |
| 8 | Nijhof et al., 2011 [67] | CDC-1994 | Netherlands | C&A (P) | Physician | 73,636 (-/-) | 81 (-/-) | 0.11 |
| 9 | van't Leven et al., 2010 [68] | CDC-1994 | Netherlands | General (C) | Interview | 9,062 (4,169/4,893) | 89 (-/-) | 0.98 |
| 10 | Cho et al., 2009 [69] | CDC-1994 | UK | General (P) | I&M | 2,459 (700/1,759) | 51 (14/37) | 2.07 |
| 11 | | CDC-1994 | Brazil | | | 3,914 (794/3,120) | 63 (13/50) | 1.61 |
| 12 | Kim S, 2008 [70] | CDC-1994 | Korea | General (C) | Interview | 1,644 (815/829) | 1 (1/0) | 0.06 |
| 13 | Kim SH, 2008 [71] | CDC-1994 | Korea | Sp (C) | Interview | 971 (637/334) | 4 (0/4) | 0.41 |
| 14 | Reeves et al., 2007 [72] | CDC-1994 | USA | General (C) | I&M | 5,623 (-/-) | 113 (-/-) | 2.01 |
| 15 | Njoku et al., 2007 [50] | CDC-1994 | Nigeria | General (C) | I&M | 1,087 (455/599) | 3 (0/3) | 0.28 |
| 16 | Paralikar et al., 2007 [73] | N/A | India | General (P) | Interview | 1,874 (-/-) | 94 (-/-) | 5.02 |
| 17 | Rimes et al., 2007 [74] | CDC-1994 | UK | C&A (C) | Interview | 842 (-/-) | 4 (-/-) | 0.48 |
| 18 | Kim CH. et al., 2005 [55] | CDC-1994 | Korea | General (P) | I&M | 1,648 (591/1,057) | 10 (2/8) | 0.61 |
| 19 | Furberg et al., 2005 [75] | CDC-1994 | USA | General (C) | Interview | 4,591 (1,607/2,984) | 126 (45/81) | 2.74 |
| 20 | Evengard et al., 2005 [76] | CDC-1994 | Sweden | General (C) | Interview | 31405 (15387/16017) | 732 (140/592) | 2.33 |
| 21 | Yiu and Qiu, 2005 [77] | CDC-1994 | China | General (C) | Interview | 1,013 (341/672) | 65 (13/43) | 6.42 |
| 22 | Huibers et al., 2004 [78] | CDC-1994 | Netherlands | Sp (C) | Interview | 5,499 (-/-) | 199 (144/55) | 3.62 |
| 23 | Jones et al., 2004 [79] | CDC-1994 | USA | C&A (C) | I&M | 8,586 (-/-) | 29 (-/-) | 0.34 |
| 24 | Farmer et al., 2004 [80] | CDC-1994 | UK | C&A (C) | Interview | 1,468 (-/-) | 35 (9/26) | 2.38 |
| 25 | Chalder et al., 2003 [81] | CDC-1994 | UK | C&A (C) | Interview | 4,240 (-/-) | 8 (-/-) | 0.19 |
| 26 | Reyes et al., 2003 [54] | CDC-1994 | USA | General (C) | I&M | 7,162 (-/-) | 43 (7/36) | 0.60 |
| 27 | McCauley et al., 2002 [82] | CDC-1994 | USA | Sp (C) | I&M | 799 (-/-) | 18 (-/-) | 2.25 |
| 28-1 | Líndal et al., 2002 [34] | CDC-1994 | Iceland | General (C) | Interview | 2,520 (1,056/1,415) | 54 (12/42) | 2.14 |
| 28-2 | | Holmes | | | | | 0 (-/-) | 0.02 |
| 28-3 | | Oxford | | | | | 94 (20/74) | 3.73 |
| 28-4 | | Australian | | | | | 192 (28/164) | 7.62 |
| 29 | Kim CH. et al., 2000 [83] | CDC-1994 | Korea | General (P) | I&M | 988 (-/-) | 12 (-/-) | 1.21 |
| 30 | Ji JD, 2000 [84] | CDC-1994 | Korea | General (P) | I&M | 1,526 (906/620) | 31 (13/18) | 2.03 |
| 31 | Ji JD, 1999 [85] | CDC-1994 | Korea | General (P) | I&M | 530 (327/203) | 10 (5/5) | 1.89 |
| 32 | Jason et al., 1999 [53] | CDC-1994 | USA | General (C) | I&M | 28673 (16057/12329) | 119 (67/52) | 0.42 |
| 33 | Jason et al., 1998 [86] | CDC-1994 | USA | Sp (P) | I&M | 3400 (-/-) | 37 (-/-) | 1.09 |
| 34 | Steele et al., 1998 [87] | CDC-1994 | USA | General (C) | Interview | 14,627 (7,198/7,409) | 33 (8/25) | 0.23 |
| 35 | Fukuda et al., 1997 [88] | CDC-1994 | UK | General (C) | Interview | 1,689 (748/941) | 58 (-/-) | 3.43 |
| 36-1 | Wessely et al., 1997 [16] | CDC-1994 | UK | General (P) | I&M | 1,985 (-/-) | 52 (-/-) | 2.62 |
| 36-2 | | Oxford | | | | | 44 (-/-) | 2.22 |
| 36-3 | | Australian | | | | | 28 (-/-) | 1.41 |
| 36-4 | | Holmes | | | | | 24 (-/-) | 1.21 |
| 37 | Versluis et al., 1997 [89] | Holmes | Netherlands (R) | General (P) | MR | 23,000 (-/-) | 25 (-/-) | 0.11 |
| 38 | Minowa and Jiamo, 1996 [90] | CDC-1994 | Japan | General (P) | Interview | 65,500 (-/-) | 393 (129/244) | 0.60 |
| 39 | Lawrie and Pelosi, 1995 [60] | Oxford | UK | General (C) | Interview | 695 (322/373) | 4 (2/2) | 0.58 |
| 40 | Buchwald and Umali, 1995 [91] | Holmes | USA | General (C) | I&M | 3,066 (1,226/1,839) | 3 (1/2) | 0.1 |
| 41 | Jason et al., 1995 [92] | Holmes | USA | General (C) | I&M | 913 (367/501) | 2 (0/2) | 0.22 |
| 42 | Jason et al., 1993 [93] | Holmes | USA | Sp (P) | Interview | 1,474 (-/-) | 11 (2/9) | 0.75 |
| 43-1 | Bates et al., 1993 [39] | Oxford | USA | General (P) | I&M | 995 (-/-) | 4 (-/-) | 0.40 |
| 43-2 | | Holmes | | | | | 3 (-/-) | 0.30 |
| 43-3 | | Australian | | | | | 10 (-/-) | 1.01 |
| 44 | Price et al., 1992 [33] | Holmes | USA | General (C) | Interview | 13538 (5556/7982) | 1 (0/1) | 0.01 |
| 45 | Ho-Yen and Mc, 1991 [36] | PVES | UK | General (P) | Physician | 218,993 (-/-) | 293 (102/187) | 0.13 |
| 46 | Lloyd et al., 1990 [25] | Australian | Australia | General (C) | Physician | 218,993 (-/-) | 42 (-/-) | 0.04 |
| C&A, Children and adolescents. Sp, specific population. I&M, interview and medical test. MR, medical record. PVES, postviral exhaustion syndrome. (R), retrospective studies, otherwise, prospective studies. (C), community. (P), primary care. | | | | | | | | |
