# Supplementary material for: Systematic review and meta-analysis of the prevalence of chronic fatigue syndrome/myalgic encephalomyelitis (CFS/ME)
Source: J Transl Med. 2020 Feb 24;18:100. doi: 10.1186/s12967-020-02269-0 (PMC7038594; doi:10.1186/s12967-020-02269-0)
Supplement: Supplementary file 3 — Additional file 3: Meta-analysis of the CFS/ME prevalence for children and adolescents (A) and specific populations (B). [file 12967_2020_2269_MOESM3_ESM.pptx]

## Slide 1
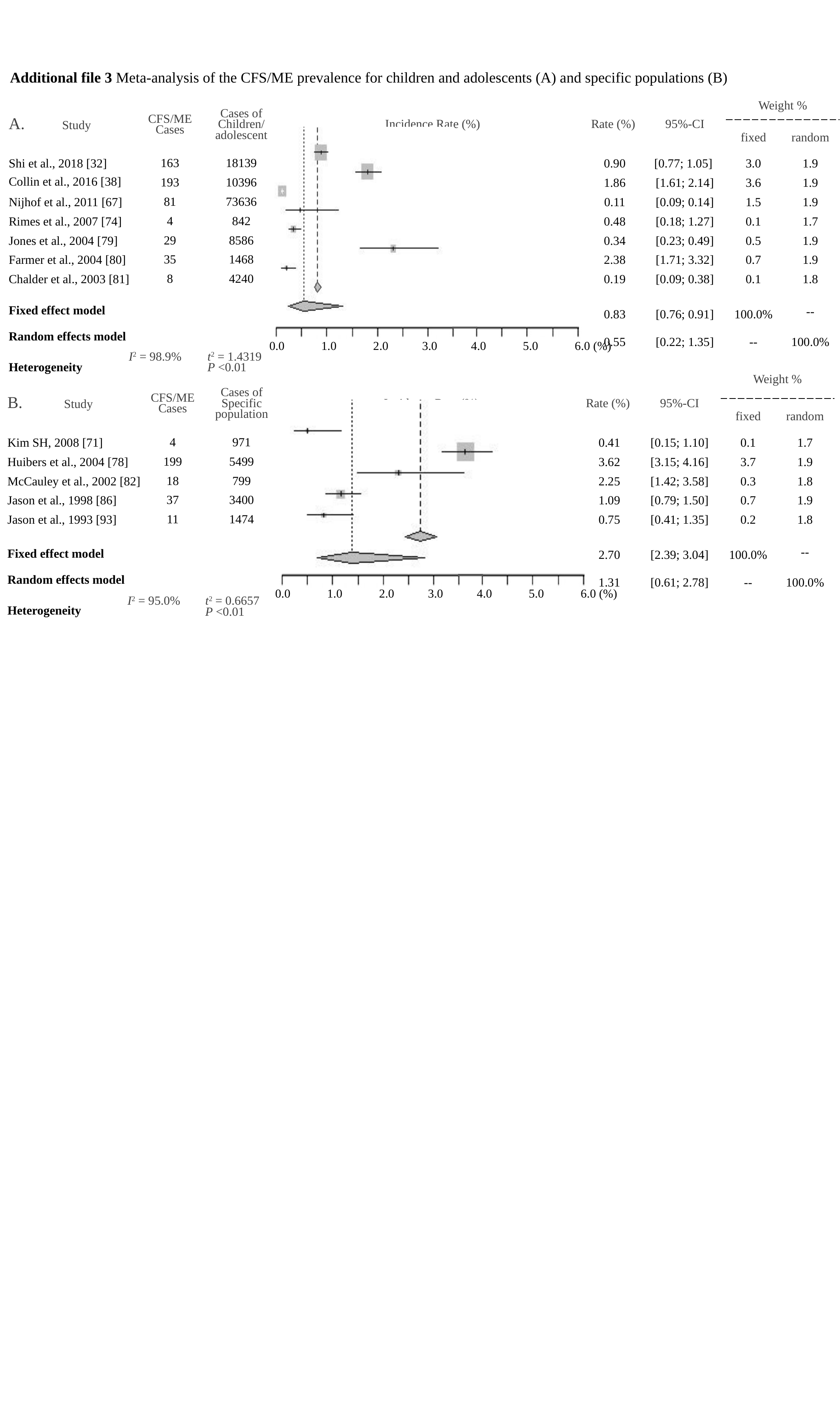

Additional file 3 Meta-analysis of the CFS/ME prevalence for children and adolescents (A) and specific populations (B)
| A. Study | | CFS/ME Cases | Cases of Children/ adolescent | Incidence Rate (%) | Rate (%) | 95%-CI | Weight % | |
| --- | --- | --- | --- | --- | --- | --- | --- | --- |
| | | | | | | | fixed | random |
| Shi et al., 2018 [32] | | 163 | 18139 | | 0.90 | [0.77; 1.05] | 3.0 | 1.9 |
| Collin et al., 2016 [38] | | 193 | 10396 | | 1.86 | [1.61; 2.14] | 3.6 | 1.9 |
| Nijhof et al., 2011 [67] | | 81 | 73636 | | 0.11 | [0.09; 0.14] | 1.5 | 1.9 |
| Rimes et al., 2007 [74] | | 4 | 842 | | 0.48 | [0.18; 1.27] | 0.1 | 1.7 |
| Jones et al., 2004 [79] | | 29 | 8586 | | 0.34 | [0.23; 0.49] | 0.5 | 1.9 |
| Farmer et al., 2004 [80] | | 35 | 1468 | | 2.38 | [1.71; 3.32] | 0.7 | 1.9 |
| Chalder et al., 2003 [81] | | 8 | 4240 | | 0.19 | [0.09; 0.38] | 0.1 | 1.8 |
| Fixed effect model | | | | | 0.83 | [0.76; 0.91] | 100.0% | -- |
| Random effects model | | | | | 0.55 | [0.22; 1.35] | -- | 100.0% |
| Heterogeneity | I2 = 98.9% | | t2 = 1.4319 P <0.01 | | | | | |
 0.0 1.0 2.0 3.0 4.0 5.0 6.0 (%)
| B. Study | | CFS/ME Cases | Cases of Specific population | Incidence Rate (%) | Rate (%) | 95%-CI | Weight % | |
| --- | --- | --- | --- | --- | --- | --- | --- | --- |
| | | | | | | | fixed | random |
| Kim SH, 2008 [71] | | 4 | 971 | | 0.41 | [0.15; 1.10] | 0.1 | 1.7 |
| Huibers et al., 2004 [78] | | 199 | 5499 | | 3.62 | [3.15; 4.16] | 3.7 | 1.9 |
| McCauley et al., 2002 [82] | | 18 | 799 | | 2.25 | [1.42; 3.58] | 0.3 | 1.8 |
| Jason et al., 1998 [86] | | 37 | 3400 | | 1.09 | [0.79; 1.50] | 0.7 | 1.9 |
| Jason et al., 1993 [93] | | 11 | 1474 | | 0.75 | [0.41; 1.35] | 0.2 | 1.8 |
| Fixed effect model | | | | | 2.70 | [2.39; 3.04] | 100.0% | -- |
| Random effects model | | | | | 1.31 | [0.61; 2.78] | -- | 100.0% |
| Heterogeneity | I2 = 95.0% | | t2 = 0.6657 P <0.01 | | | | | |
 0.0 1.0 2.0 3.0 4.0 5.0 6.0 (%)
