# Supplementary material for: Systematic review and meta-analysis of the prevalence of chronic fatigue syndrome/myalgic encephalomyelitis (CFS/ME)
Source: J Transl Med. 2020 Feb 24;18:100. doi: 10.1186/s12967-020-02269-0 (PMC7038594; doi:10.1186/s12967-020-02269-0)
Supplement: Supplementary file 5 — Additional file 5: Meta-analysis of CFS/ME prevalence based on the Holmes (A), Australian (B), Oxford (C), and other (D) definitions. [file 12967_2020_2269_MOESM5_ESM.pptx]

## Slide 1
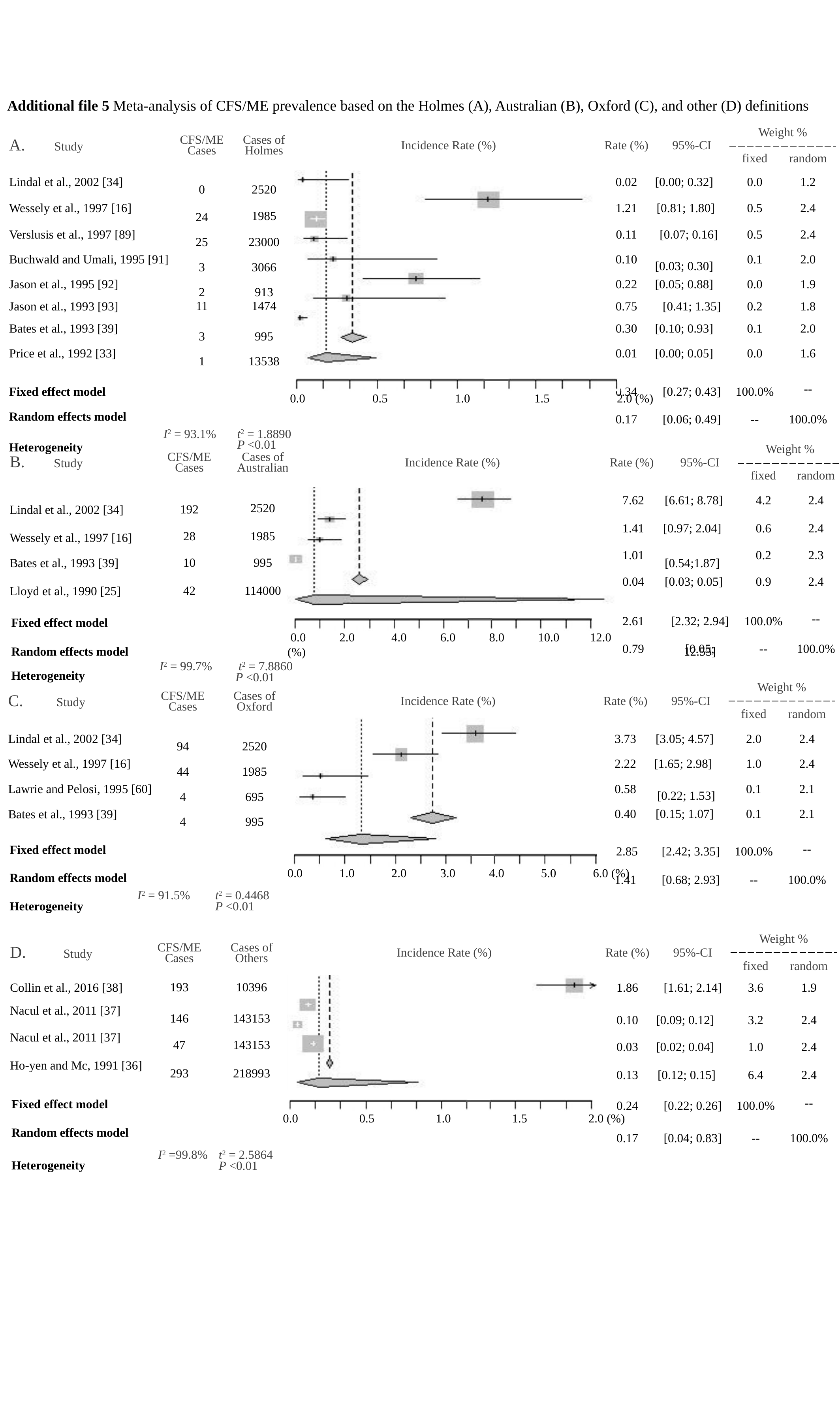

Additional file 5 Meta-analysis of CFS/ME prevalence based on the Holmes (A), Australian (B), Oxford (C), and other (D) definitions
| A. Study | | CFS/ME Cases | Cases of Holmes | Incidence Rate (%) | Rate (%) | 95%-CI | Weight % | |
| --- | --- | --- | --- | --- | --- | --- | --- | --- |
| | | | | | | | fixed | random |
| Lindal et al., 2002 [34] | | 0 | 2520 | | 0.02 | [0.00; 0.32] | 0.0 | 1.2 |
| Wessely et al., 1997 [16] | | 24 | 1985 | | 1.21 | [0.81; 1.80] | 0.5 | 2.4 |
| Verslusis et al., 1997 [89] | | 25 | 23000 | | 0.11 | [0.07; 0.16] | 0.5 | 2.4 |
| Buchwald and Umali, 1995 [91] | | 3 | 3066 | | 0.10 | [0.03; 0.30] | 0.1 | 2.0 |
| Jason et al., 1995 [92] | | 2 | 913 | | 0.22 | [0.05; 0.88] | 0.0 | 1.9 |
| Jason et al., 1993 [93] | | 11 | 1474 | | 0.75 | [0.41; 1.35] | 0.2 | 1.8 |
| Bates et al., 1993 [39] | | 3 | 995 | | 0.30 | [0.10; 0.93] | 0.1 | 2.0 |
| Price et al., 1992 [33] | | 1 | 13538 | | 0.01 | [0.00; 0.05] | 0.0 | 1.6 |
| Fixed effect model | | | | | 0.34 | [0.27; 0.43] | 100.0% | -- |
| Random effects model | | | | | 0.17 | [0.06; 0.49] | -- | 100.0% |
| Heterogeneity | I2 = 93.1% | | t2 = 1.8890 P <0.01 | | | | | |
 0.0 0.5 1.0 1.5 2.0 (%)
| B. Study | | CFS/ME Cases | Cases of Australian | Incidence Rate (%) | Rate (%) | 95%-CI | Weight % | |
| --- | --- | --- | --- | --- | --- | --- | --- | --- |
| | | | | | | | fixed | random |
| Lindal et al., 2002 [34] | | 192 | 2520 | | 7.62 | [6.61; 8.78] | 4.2 | 2.4 |
| Wessely et al., 1997 [16] | | 28 | 1985 | | 1.41 | [0.97; 2.04] | 0.6 | 2.4 |
| Bates et al., 1993 [39] | | 10 | 995 | | 1.01 | [0.54;1.87] | 0.2 | 2.3 |
| Lloyd et al., 1990 [25] | | 42 | 114000 | | 0.04 | [0.03; 0.05] | 0.9 | 2.4 |
| Fixed effect model | | | | | 2.61 | [2.32; 2.94] | 100.0% | -- |
| | | | | | 0.79 | [0.05; 12.55] | -- | 100.0% |
| Random effects model | | | | | | | | |
| Heterogeneity | I2 = 99.7% | | t2 = 7.8860 P <0.01 | | | | | |
 0.0 2.0 4.0 6.0 8.0 10.0 12.0 (%)
| C. Study | | CFS/ME Cases | Cases of Oxford | Incidence Rate (%) | Rate (%) | 95%-CI | Weight % | |
| --- | --- | --- | --- | --- | --- | --- | --- | --- |
| | | | | | | | fixed | random |
| Lindal et al., 2002 [34] | | 94 | 2520 | | 3.73 | [3.05; 4.57] | 2.0 | 2.4 |
| Wessely et al., 1997 [16] | | 44 | 1985 | | 2.22 | [1.65; 2.98] | 1.0 | 2.4 |
| Lawrie and Pelosi, 1995 [60] | | 4 | 695 | | 0.58 | [0.22; 1.53] | 0.1 | 2.1 |
| Bates et al., 1993 [39] | | 4 | 995 | | 0.40 | [0.15; 1.07] | 0.1 | 2.1 |
| Fixed effect model | | | | | 2.85 | [2.42; 3.35] | 100.0% | -- |
| Random effects model | | | | | 1.41 | [0.68; 2.93] | -- | 100.0% |
| Heterogeneity | I2 = 91.5% | | t2 = 0.4468 P <0.01 | | | | | |
 0.0 1.0 2.0 3.0 4.0 5.0 6.0 (%)
| D. Study | CFS/ME Cases | Cases of Others | Incidence Rate (%) | Rate (%) | 95%-CI | Weight % | |
| --- | --- | --- | --- | --- | --- | --- | --- |
| | | | | | | fixed | random |
| Collin et al., 2016 [38] | 193 | 10396 | | 1.86 | [1.61; 2.14] | 3.6 | 1.9 |
| Nacul et al., 2011 [37] | 146 | 143153 | | 0.10 | [0.09; 0.12] | 3.2 | 2.4 |
| Nacul et al., 2011 [37] | 47 | 143153 | | 0.03 | [0.02; 0.04] | 1.0 | 2.4 |
| Ho-yen and Mc, 1991 [36] | 293 | 218993 | | 0.13 | [0.12; 0.15] | 6.4 | 2.4 |
| Fixed effect model | | | | 0.24 | [0.22; 0.26] | 100.0% | -- |
| Random effects model | | | | 0.17 | [0.04; 0.83] | -- | 100.0% |
| Heterogeneity | I2 =99.8% | t2 = 2.5864 P <0.01 | | | | | |
 0.0 0.5 1.0 1.5 2.0 (%)
