# Supplementary material for: Systematic review and meta-analysis of the prevalence of chronic fatigue syndrome/myalgic encephalomyelitis (CFS/ME)
Source: J Transl Med. 2020 Feb 24;18:100. doi: 10.1186/s12967-020-02269-0 (PMC7038594; doi:10.1186/s12967-020-02269-0)
Supplement: Supplementary file 6 — Additional file 6: Meta-analysis of CFS/ME prevalence by interview (A) and interview with medical test (B). [file 12967_2020_2269_MOESM6_ESM.pptx]

## Slide 1
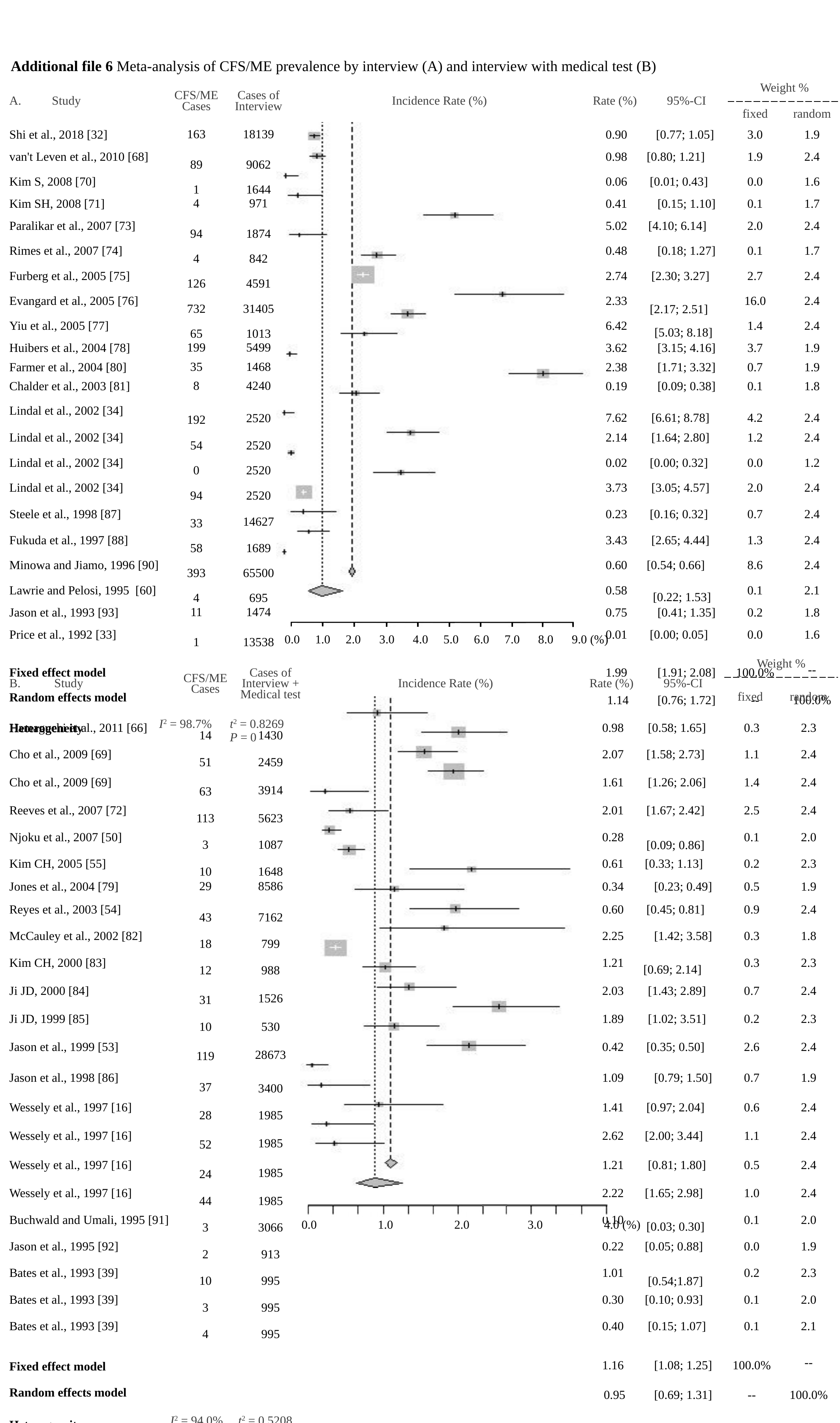

Additional file 6 Meta-analysis of CFS/ME prevalence by interview (A) and interview with medical test (B)
| A. Study | | CFS/ME Cases | Cases of Interview | Incidence Rate (%) | Rate (%) | 95%-CI | Weight % | |
| --- | --- | --- | --- | --- | --- | --- | --- | --- |
| | | | | | | | fixed | random |
| Shi et al., 2018 [32] | | 163 | 18139 | | 0.90 | [0.77; 1.05] | 3.0 | 1.9 |
| van't Leven et al., 2010 [68] | | 89 | 9062 | | 0.98 | [0.80; 1.21] | 1.9 | 2.4 |
| Kim S, 2008 [70] | | 1 | 1644 | | 0.06 | [0.01; 0.43] | 0.0 | 1.6 |
| Kim SH, 2008 [71] | | 4 | 971 | | 0.41 | [0.15; 1.10] | 0.1 | 1.7 |
| Paralikar et al., 2007 [73] | | 94 | 1874 | | 5.02 | [4.10; 6.14] | 2.0 | 2.4 |
| Rimes et al., 2007 [74] | | 4 | 842 | | 0.48 | [0.18; 1.27] | 0.1 | 1.7 |
| Furberg et al., 2005 [75] | | 126 | 4591 | | 2.74 | [2.30; 3.27] | 2.7 | 2.4 |
| Evangard et al., 2005 [76] | | 732 | 31405 | | 2.33 | [2.17; 2.51] | 16.0 | 2.4 |
| Yiu et al., 2005 [77] | | 65 | 1013 | | 6.42 | [5.03; 8.18] | 1.4 | 2.4 |
| Huibers et al., 2004 [78] | | 199 | 5499 | | 3.62 | [3.15; 4.16] | 3.7 | 1.9 |
| Farmer et al., 2004 [80] | | 35 | 1468 | | 2.38 | [1.71; 3.32] | 0.7 | 1.9 |
| Chalder et al., 2003 [81] | | 8 | 4240 | | 0.19 | [0.09; 0.38] | 0.1 | 1.8 |
| Lindal et al., 2002 [34] | | 192 | 2520 | | | | | |
| | | | | | 7.62 | [6.61; 8.78] | 4.2 | 2.4 |
| Lindal et al., 2002 [34] | | 54 | 2520 | | 2.14 | [1.64; 2.80] | 1.2 | 2.4 |
| Lindal et al., 2002 [34] | | 0 | 2520 | | 0.02 | [0.00; 0.32] | 0.0 | 1.2 |
| Lindal et al., 2002 [34] | | 94 | 2520 | | 3.73 | [3.05; 4.57] | 2.0 | 2.4 |
| Steele et al., 1998 [87] | | 33 | 14627 | | 0.23 | [0.16; 0.32] | 0.7 | 2.4 |
| Fukuda et al., 1997 [88] | | 58 | 1689 | | 3.43 | [2.65; 4.44] | 1.3 | 2.4 |
| Minowa and Jiamo, 1996 [90] | | 393 | 65500 | | 0.60 | [0.54; 0.66] | 8.6 | 2.4 |
| Lawrie and Pelosi, 1995 [60] | | 4 | 695 | | 0.58 | [0.22; 1.53] | 0.1 | 2.1 |
| Jason et al., 1993 [93] | | 11 | 1474 | | 0.75 | [0.41; 1.35] | 0.2 | 1.8 |
| Price et al., 1992 [33] | | 1 | 13538 | | 0.01 | [0.00; 0.05] | 0.0 | 1.6 |
| Fixed effect model | | | | | 1.99 | [1.91; 2.08] | 100.0% | -- |
| Random effects model | | | | | 1.14 | [0.76; 1.72] | -- | 100.0% |
| Heterogeneity | I2 = 98.7% | | t2 = 0.8269 P = 0 | | | | | |
0.0 1.0 2.0 3.0 4.0 5.0 6.0 7.0 8.0 9.0 (%)
| B. Study | | CFS/ME Cases | Cases of Interview + Medical test | Incidence Rate (%) | Rate (%) | 95%-CI | Weight % | |
| --- | --- | --- | --- | --- | --- | --- | --- | --- |
| | | | | | | | fixed | random |
| Hamaguchi et al., 2011 [66] | | 14 | 1430 | | 0.98 | [0.58; 1.65] | 0.3 | 2.3 |
| Cho et al., 2009 [69] | | 51 | 2459 | | 2.07 | [1.58; 2.73] | 1.1 | 2.4 |
| Cho et al., 2009 [69] | | 63 | 3914 | | 1.61 | [1.26; 2.06] | 1.4 | 2.4 |
| Reeves et al., 2007 [72] | | 113 | 5623 | | 2.01 | [1.67; 2.42] | 2.5 | 2.4 |
| Njoku et al., 2007 [50] | | 3 | 1087 | | 0.28 | [0.09; 0.86] | 0.1 | 2.0 |
| Kim CH, 2005 [55] | | 10 | 1648 | | 0.61 | [0.33; 1.13] | 0.2 | 2.3 |
| Jones et al., 2004 [79] | | 29 | 8586 | | 0.34 | [0.23; 0.49] | 0.5 | 1.9 |
| Reyes et al., 2003 [54] | | 43 | 7162 | | 0.60 | [0.45; 0.81] | 0.9 | 2.4 |
| McCauley et al., 2002 [82] | | 18 | 799 | | 2.25 | [1.42; 3.58] | 0.3 | 1.8 |
| Kim CH, 2000 [83] | | 12 | 988 | | 1.21 | [0.69; 2.14] | 0.3 | 2.3 |
| Ji JD, 2000 [84] | | 31 | 1526 | | 2.03 | [1.43; 2.89] | 0.7 | 2.4 |
| Ji JD, 1999 [85] | | 10 | 530 | | 1.89 | [1.02; 3.51] | 0.2 | 2.3 |
| Jason et al., 1999 [53] | | 119 | 28673 | | 0.42 | [0.35; 0.50] | 2.6 | 2.4 |
| Jason et al., 1998 [86] | | 37 | 3400 | | 1.09 | [0.79; 1.50] | 0.7 | 1.9 |
| Wessely et al., 1997 [16] | | 28 | 1985 | | 1.41 | [0.97; 2.04] | 0.6 | 2.4 |
| Wessely et al., 1997 [16] | | 52 | 1985 | | 2.62 | [2.00; 3.44] | 1.1 | 2.4 |
| Wessely et al., 1997 [16] | | 24 | 1985 | | 1.21 | [0.81; 1.80] | 0.5 | 2.4 |
| Wessely et al., 1997 [16] | | 44 | 1985 | | 2.22 | [1.65; 2.98] | 1.0 | 2.4 |
| Buchwald and Umali, 1995 [91] | | 3 | 3066 | | 0.10 | [0.03; 0.30] | 0.1 | 2.0 |
| Jason et al., 1995 [92] | | 2 | 913 | | 0.22 | [0.05; 0.88] | 0.0 | 1.9 |
| Bates et al., 1993 [39] | | 10 | 995 | | 1.01 | [0.54;1.87] | 0.2 | 2.3 |
| Bates et al., 1993 [39] | | 3 | 995 | | 0.30 | [0.10; 0.93] | 0.1 | 2.0 |
| Bates et al., 1993 [39] | | 4 | 995 | | 0.40 | [0.15; 1.07] | 0.1 | 2.1 |
| Fixed effect model | | | | | 1.16 | [1.08; 1.25] | 100.0% | -- |
| Random effects model | | | | | 0.95 | [0.69; 1.31] | -- | 100.0% |
| Heterogeneity | I2 = 94.0% | | t2 = 0.5208 P <0.01 | | | | | |
0.0 1.0 2.0 3.0 4.0 (%)
