# Supplementary material for: Systematic review and meta-analysis of the prevalence of chronic fatigue syndrome/myalgic encephalomyelitis (CFS/ME)
Source: J Transl Med. 2020 Feb 24;18:100. doi: 10.1186/s12967-020-02269-0 (PMC7038594; doi:10.1186/s12967-020-02269-0)
Supplement: Supplementary file 7 — Additional file 7: Meta-analysis of prevalence studies with diagnosis by physician determination (A) and review of medical records (B). [file 12967_2020_2269_MOESM7_ESM.pptx]

## Slide 1
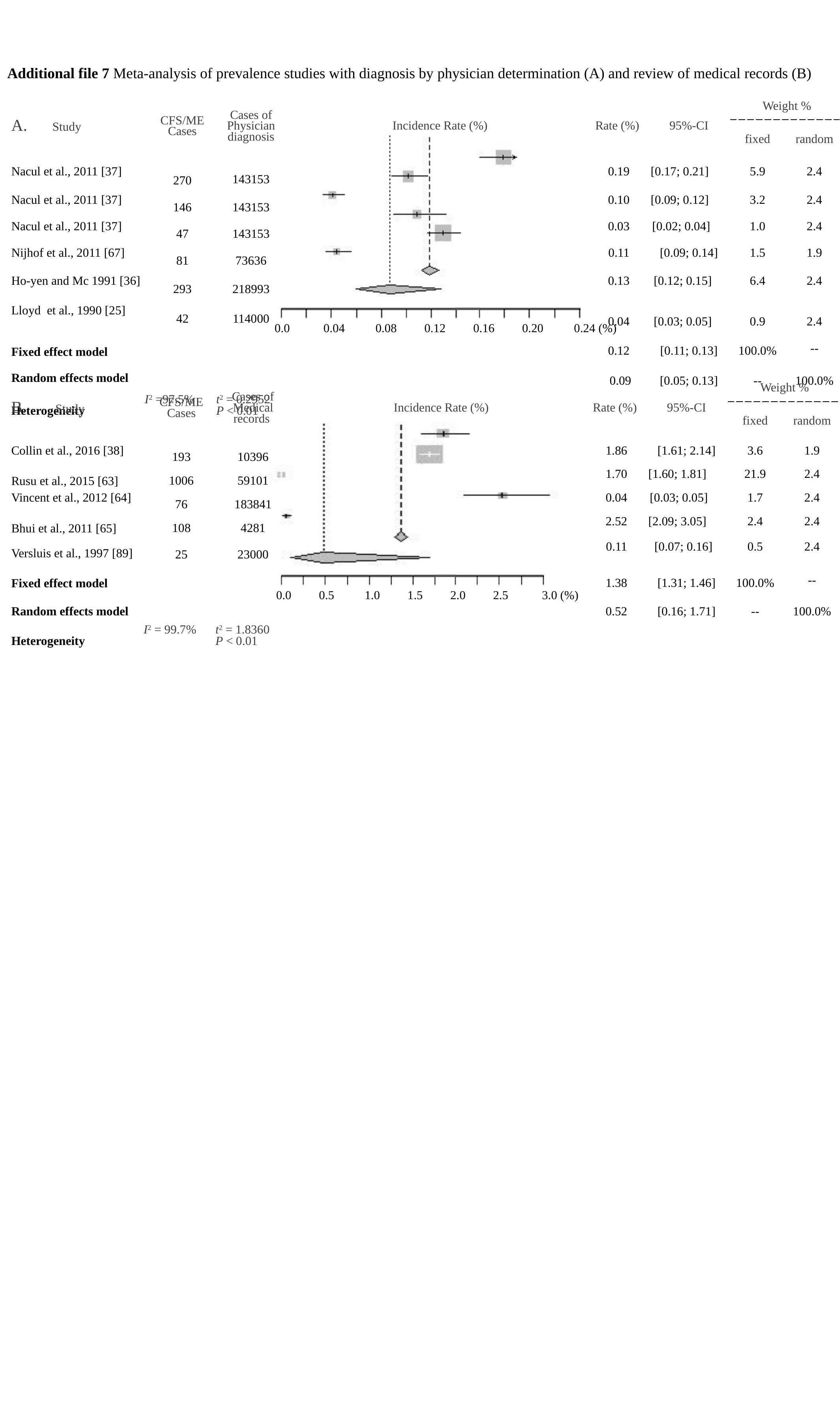

Additional file 7 Meta-analysis of prevalence studies with diagnosis by physician determination (A) and review of medical records (B)
| A. Study | | CFS/ME Cases | Cases of Physician diagnosis | Incidence Rate (%) | Rate (%) | 95%-CI | Weight % | |
| --- | --- | --- | --- | --- | --- | --- | --- | --- |
| | | | | | | | fixed | random |
| Nacul et al., 2011 [37] | | 270 | 143153 | | 0.19 | [0.17; 0.21] | 5.9 | 2.4 |
| Nacul et al., 2011 [37] | | 146 | 143153 | | 0.10 | [0.09; 0.12] | 3.2 | 2.4 |
| Nacul et al., 2011 [37] | | 47 | 143153 | | 0.03 | [0.02; 0.04] | 1.0 | 2.4 |
| Nijhof et al., 2011 [67] | | 81 | 73636 | | 0.11 | [0.09; 0.14] | 1.5 | 1.9 |
| Ho-yen and Mc 1991 [36] | | 293 | 218993 | | 0.13 | [0.12; 0.15] | 6.4 | 2.4 |
| Lloyd et al., 1990 [25] | | 42 | 114000 | | 0.04 | [0.03; 0.05] | 0.9 | 2.4 |
| Fixed effect model | | | | | 0.12 | [0.11; 0.13] | 100.0% | -- |
| Random effects model | | | | | 0.09 | [0.05; 0.13] | -- | 100.0% |
| Heterogeneity | I2 =97.5% | | t2 = 0.2952 P < 0.01 | | | | | |
0.0 0.04 0.08 0.12 0.16 0.20 0.24 (%)
| B. Study | | CFS/ME Cases | Cases of Medical records | Incidence Rate (%) | Rate (%) | 95%-CI | Weight % | |
| --- | --- | --- | --- | --- | --- | --- | --- | --- |
| | | | | | | | fixed | random |
| Collin et al., 2016 [38] | | 193 | 10396 | | 1.86 | [1.61; 2.14] | 3.6 | 1.9 |
| Rusu et al., 2015 [63] | | 1006 | 59101 | | 1.70 | [1.60; 1.81] | 21.9 | 2.4 |
| Vincent et al., 2012 [64] | | 76 | 183841 | | 0.04 | [0.03; 0.05] | 1.7 | 2.4 |
| Bhui et al., 2011 [65] | | 108 | 4281 | | 2.52 | [2.09; 3.05] | 2.4 | 2.4 |
| Versluis et al., 1997 [89] | | 25 | 23000 | | 0.11 | [0.07; 0.16] | 0.5 | 2.4 |
| Fixed effect model | | | | | 1.38 | [1.31; 1.46] | 100.0% | -- |
| Random effects model | | | | | 0.52 | [0.16; 1.71] | -- | 100.0% |
| Heterogeneity | I2 = 99.7% | | t2 = 1.8360 P < 0.01 | | | | | |
0.0 0.5 1.0 1.5 2.0 2.5 3.0 (%)
